# Micro-Doppler Feature Extraction of Inverse Synthetic Aperture Imaging Laser Radar Using Singular-Spectrum Analysis

**DOI:** 10.3390/s18103303

**Published:** 2018-10-01

**Authors:** Mingzhe Zhu, Xianda Zhou, Bo Zang, Baisheng Yang, Mengdao Xing

**Affiliations:** 1 School of Electronic Engineering, Xidian University, Xi’an 710126, China; zhumz@mail.xidian.edu.cn (M.Z.); zhouxianda999@hotmail.com (X.Z.); xmd@xidian.edu.cn (M.X.); 2 Beijing Aerospace Automatic Control Institute, Beijing 100000, China; tfybs@163.com

**Keywords:** inverse synthetic aperture imaging laser radar, micro-Doppler effect, singular-spectrum analysis

## Abstract

Different from microwave radar, laser radar could be more sensitive to the micro-Doppler (m-D) effect due to its wave length. This limits the application of conventional methods, such as time–frequency based approach, since the processing needs a receiver with much higher sampling frequency than microwave radar. In this paper, a micro-Doppler feature extraction algorithm is proposed for the inverse synthetic aperture imaging laser radar (ISAIL). Singular-spectrum analysis (SSA) is employed for separation and reconstruction of the micro-Doppler and rigid body signal. Clear ISAIL image is obtained by minimum entropy criteria after echo signal decomposition. After theoretical derivation, the computation efficiency and ability of the proposed method is proved by the results of simulation and real data of An-26.

## 1. Introduction

For the radar echo signal, vibration and rotation of target or the target’s structure may cause frequency modulation, which is called the micro-Doppler (m-D) effect. Rotation of propellers or rotor wings on plane, surface vibration caused by engine, and swinging arms when human walk may cause the m-D effect. Up to now, varies studies have been done on m-D effect. However, most are limited to the microwave approach only. Time–frequency analysis can be used in feature extraction or parameter estimation of m-D component [1,2,3,4]. Adaptive chirplet representation divides target’s echo of rigid parts and moving parts into different parameter spaces to separate two kind of signal [5]. The m-D components can be restrained or separated with windowed time–frequency analysis and Radon transform [6].

In the relevant method, the extraction of m-D features based on singular value decomposition (SVD) has attracted extensive attention due to its advantages in feature representation and computational complexity. Related results analyze and extract the characteristics of m-D effect from different aspects [7,8,9,10].

The m-D effect on laser radar has increased importance since the development of coherent laser radar [11,12]. ISAIL is such a coherent laser radar; the micron-size working wavelength makes a sensitive detection of the m-D effect of target. It may mainly lead to two kinds of results for ISAIL imaging. First, m-D effect would bring doppler broadening, which causes the banded interference image obtains from distance-doppler domain. This is troublesome for feature extraction and understanding of the image. Second, the m-D components in the echo signal have abundant motion information of moving parts, which means a new approach of features analysis of target. Separating two different types of signals and ensuring their integrity to meet the demand of ISAIL imaging and moving parts dynamic feature analysis have become important research topics.

The m-D effect of ISAIL has the characteristics of coherent laser signal. For wavelengths far lower than microwave, ISAIL could be more sensitive to the m-D effect and obtain more m-D components. That may be seen as the interference to the imaging, or more information of moving parts. Furthermore, different from microwave approach, higher frequency of ISAIL’s m-D signal needs receiver with higher sampling frequency, which is not commonly satisfied.

For these characteristics of m-D effect of ISAIL, the m-D analysis methods applied to microwave image radar are restricted. For example, chirplet decomposition has a high computational complexity. Existing time–frequency methods usually take low frequency m-D signals for simulation, while the sampling frequency of receiver system cannot meet the demand of real data. That makes the time–frequency method difficult to get the correct m-D information. Meanwhile, it is difficult to estimate their m-D frequency by time–frequency analysis when multiple scattering points of different vibration modes exist simultaneously in one range cell.

For these problems, this paper propose a m-D effect analysis method based on Singular-Spectrum Analysis (SSA). Echo signal is decomposed with SSA after translational motion compensation and phase error correction. Then, the m-D component from moving parts is separated with component from rigid parts. By minimum entropy criteria, clear ISAIL image is obtained. High computation efficiency and ability for m-D effect is proved by the results of simulation and real data from An-26.

The rest of this paper is organized as follows. The m-D model of ISAIL is discussed in Section 2 and a brief introduction of SSA algorithm is given in Section 3. The complete description of proposed technique is given in Section 4. Results of numerical simulations and real data tests are presented in Section 5 and concluding remarks in Section 6.

## 2. Model of ISAIL m-D Effect

**Figure 1:**
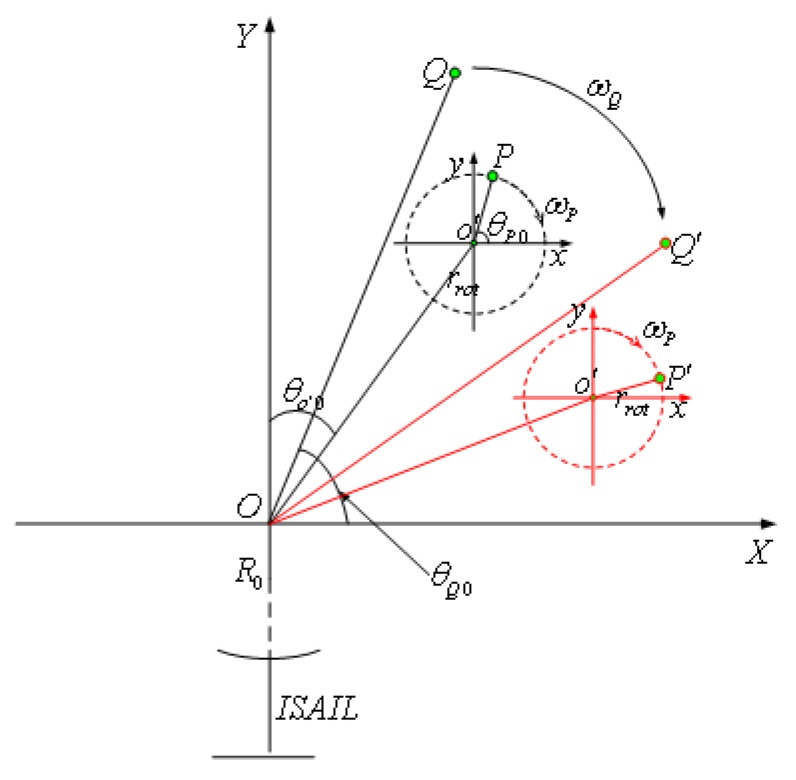
Cumulative probability function as a STFT result.

The model of ISAIL m-D effect can be described as Figure 1.

*Q* and *P* are rigid and rotation scattering point of target. Echo signal after range compression is defined as:

(1) $$S\left(f_{r},t_{m}\right)=AT_{p}c\cdot \sin\left[T_{p}\left(f_{r}+2\frac{\gamma }{c}R_{\Delta }\left(t_{m}\right)\right)\right]\exp\left(-j2\pi \frac{2}{\lambda }R_{\Delta }\left(t_{m}\right)\right)$$

where *A* is the amplitude of echo signal, $T_{p}$ is the pulse width, γ is the modulation rate, *c* is the velocity of light, λ is the wave length of carrier frequency and $R_{\Delta }\left(t_{m}\right)=R-R_{0}$ is the distance difference for scattering point to radar displaced phase center and reference point.

For rigid scattering point *Q*

(2) $$\begin{array}{cc} R_{\Delta Q}\left(t_{m}\right) & =\sqrt{R_{0}^{2}+r_{Q}^{2}-2R_{0}r_{Q}\cos\left(\theta \left(t_{m}\right)+\theta_{Q0}+\frac{\pi }{2}\right)}\\ & \approx r_{Q}\sin\left(\theta_{Q}\left(t_{m}\right)+\theta_{Q0}\right) \end{array}$$

where $r_{Q}$ is the distance from rigid rotation center to point *Q*, $\theta_{Q0}$ is the angle between *X* axis and OQ in coordinate XOY and $\theta_{Q}\left(t_{M}\right)=\omega t_{m}$. Based on the assumption of the uniform circular motion for point *Q*, it is centered at *O* with angular velocity ω in the coherent accumulative time.

Since the accumulation on cross-range is very small here, it has $\cos(\theta_{Q}\left(t_{m}\right))\approx 1$, $\sin(\theta_{Q}\left(t_{m}\right))\approx 0$. Doppler frequency of *Q* is given by:

(3) $$f_{d}Q=\frac{2}{\lambda }\frac{dR_{\Delta Q}\left(t_{m}\right)}{dt_{m}}\approx \frac{2}{\lambda }\omega_{Q}x_{Q0}$$

For high-speed rotating scattering point *P* on target, while it has the same rotation center to point *O* as *Q*, another rotation with center point $o^{\prime }$ of moving parts makes it different. As in Figure 1, it has

(4) $$R_{\Delta P}\approx r_{o^{\prime }}\sin\left(\theta_{o^{\prime }}\left(t_{m}\right)+\theta_{o^{\prime }0}\right)+r_{rot}\sin\left(\theta_{p}\left(t_{m}\right)+\theta_{P0}\right)$$

where $r_{o}^{\prime }$ is the distance from $o^{\prime }$ to *O*, and $\theta_{o^{\prime }0}$ is the angle between *X* axis and $Oo^{\prime }$ at zero time. They have $\theta_{o^{\prime }}\left(t_{m}\right)=\omega t_{m}$. Similarly, $r_{rot}$ is the radius of rotation of *P*, and $\theta_{P0}$ is the angle between *X* axis and $o^{\prime }P$ at zero time, which are related as $\theta_{P}\left(t_{m}\right)=\omega_{P}t_{m}$. In comparison to *Q*, one more rotation of *P* introduces the sinusoidal modulation term to its doppler frequency.

(5) $$\begin{array}{cc} f_{d}p & =\frac{2}{\lambda }\frac{dR_{\Delta Q}\left(t_{m}\right)}{dt_{m}}\\ & =\frac{2}{\lambda }\frac{d[r_{o^{\prime }}\sin\left(\theta_{o^{\prime }}\left(t_{m}\right)+\theta_{o^{\prime }0}\right)+r_{rot}\sin\left(\theta_{p}\left(t_{m}\right)+\theta_{P0}\right))]}{dt_{m}}\\ & \approx \frac{2}{\lambda }\left[x_{o^{\prime }}\omega_{Q}+r_{rot}\cos\left(\theta_{p}\left(t_{m}\right)+\theta_{P0}\right)\omega_{P}\right] \end{array}$$

which is called m-D information.

In coherent accumulative time of the model above, rigid parts are seen as superposition of sinusoidal waves in ISAIL’s cross-range. Then, the moving parts are added to the modulation. When there are Nr scattering points in rigid parts, and Nm in moving parts, echo signal could be written as

(6) $$\left\{\begin{array}{cc} s & =s_{1}+s_{2}\\ s_{1} & =\sum_{Nr}^{p=1}A_{1p}\exp\left(j2\pi f_{1p}t\right)\\ s_{2} & =\sum_{Nm}^{p=1}A_{2q}\exp\left(j2\pi \left(f_{2q}t+\lambda_{q}\sin\left(\omega_{q}t\right)\right)\right) \end{array}\right.$$

where $s_{1}$ represents the components from rigid parts, and $A_{1p}$ and $f_{1p}$ are the amplitude and frequency of cross-range doppler signal for *p*th scattering point. $s_{2}$, $A_{2q}$ and $f_{2q}$ have the same meaning for moving parts, and $\lambda_{q}\sin\left(\omega_{q}t\right)$ is the m-D information. Feature extraction of m-D is to do the lossless separation for $s_{1}$ and $s_{2}$ as far as possible.

## 3. Singular Spectrum Analysis

As a special form of principal component analysis, singular spectrum analysis (SSA) is nonparametric method developed in recent years [13,14,15,16]. Detailed description and application in time series analysis can be found in Ref. [17].

Basic SSA can be described as four steps in two stages.

Step 1: Embedding. Reshape the one-dimensional time series with proper window function representation to form the trajectory matrix.

Step 2: Singular Value Decomposition (SVD). Perform the singular value decomposition (SVD) of the trajectory matrix and take the eigenvalues in the decreasing order of magnitude. Structure series of elementary matrices to have rank 1 by the corresponding eigenvectors.

The first two steps come down to the decomposition stage. Its purpose is to decompose the time series into the sum of independent components, such as trend, vibration and noise. The window length is the only variable in this stage.

Step 3: Grouping. Group the eigenvalues obtained from SVD. Then, add the bi-orthogonal matrices corresponding to the same set of eigenvalues. Decompose the autocovariance matrices of the trajectory matrices into different resultant matrices according to the different components in the original time series.

Step 4: Reconstruction. Restore each resultant matrix to the corresponding structural component. The sum of the structural component is the original time series.

Steps 3 and 4 are called stage of reconstruction.

In the process of ISAIL imaging, the orientation Doppler of the target rigid parts can be regarded as the superposition of multiple single frequency signals, presenting the trend series. Vibration series are obtained from moving parts. The goal of SSA is to separate the additive components such as trend components, vibration components, and noise components in the original time series. Therefore, from a theoretical view, SSA is suitable for the problem of ISAIL imaging m-D feature extraction.

## 4. ISAIL Micro-Doppler Feature Extraction Algorithm Based on Singular Spectrum Analysis

Suppose *N* is the number of ISAIL echoes, and the number of distance units is *M*. Echo signal is discretized along the cross-range

(7) $$S_{i}={\left[s_{i,0},\cdots ,s_{i,j},\cdots ,s_{i,M-1}\right]}^{T}$$

where i=0,1,⋯,N−1 represents the *i*th distance unit and j=0,1,⋯,m−1 represents the *j*th orientation unit. For $S_{i}$, apply SSA theory described in Section 3 to the ISAIL m-D feature extraction processing.

### 4.1. Embedding

Select window function with length *L*, where 1<L<M and K=M−L+1. The trajectory vector is

(8) $$X_{p}={\left[s_{i,p-1},\cdots ,s_{i,p+L-2}\right]}^{T}$$

${\left(•\right)}^{T}$ represents vector transposition, p=1,⋯,k. Then, the trajectory matrix is

(9) $$X=\left[X_{1},\cdots ,X_{K}\right]=\left[\begin{array}{cccc} s_{i,0} & s_{i,1} & \ldots & s_{i,K-1}\\ s_{i,1} & s_{i,2} & \ldots & s_{i,K}\\ \vdots & \vdots & \ddots & \vdots \\ s_{i,L} & s_{i,L+1} & \ldots & s_{i,M-1} \end{array}\right]$$

Here, only *L* is variable. In ISAIL imaging, since the Doppler of the rigid body scattering point exhibits a trend component with respect to moving part, to separate the micro-Doppler component from the main signal of target, the larger *L* should be selected [13], generally around M/2.

### 4.2. SVD

Make $R={\mathrm{XX}}^{T}$; then, the eigenvalues of the auto-covariance matrix of the trajectory matrix is obtained by SVD and are arranged in descending order:

(10) $$\lambda_{1}\geq \lambda_{2}\geq \cdots \geq \lambda_{L}\geq 0$$

Define the eigenvectors corresponding to these feature eigenvalues as $U_{1},U_{2},\cdots ,U_{L}$.

For $d=max\left(i\right),\;\lambda_{i}>0$, set vector

(11) $$V_{q}=\frac{X^{T}U_{q}}{\sqrt{\lambda_{q}}}\;q=1,\cdots ,d$$

Trajectory matrix X can be expressed as

(12) $$X=X_{1}+\cdots +X_{q}+\cdots +X_{d}$$

$X_{q}=\sqrt{\lambda_{q}}U_{q}V_{q}^{T}$, is an elementary matrix with rank 1.

### 4.3. Grouping

The process of m-D feature extraction can be simplified into the separation of trend component and vibration component. In the autocovariance matrix of trajectory matrix, eigenvalue corresponding to the Doppler of the rigid parts scattering point is usually much larger than the eigenvalue corresponding to the Doppler scattering point of the moving part.

**Figure 2:**
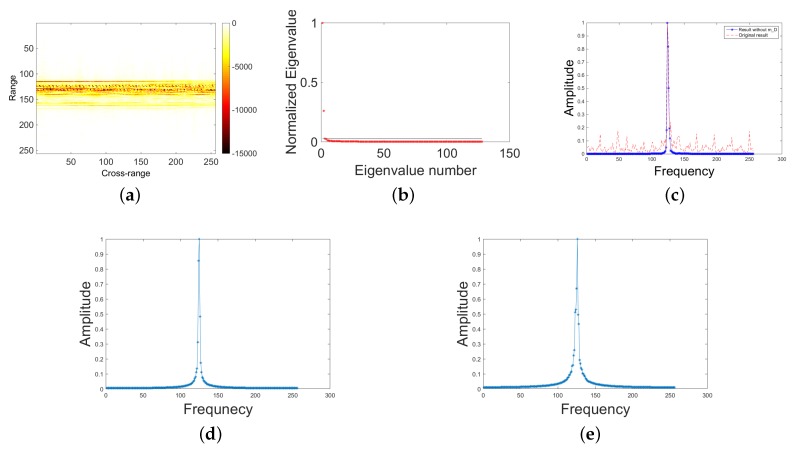
Real data from An-26: (**a**) results after the processing of distance pulse pressure, envelope alignment and phase correction; (**b**) eigenvalues of the self-covariance sorted by value; (**c**) pulse compression result; (**d**) the principal component corresponding to the first eigenvalue; and (**e**) the principal component corresponding to the second eigenvalue.

The data used in Figure 2 are the signal analysis of a certain range cell of the real ISAIL data of An-26 aircraft. Figure 2a shows the results after the processing of distance pulse pressure, envelope alignment and phase correction. It can be seen that the 118–142 range cell has obvious m-D features. The first two steps of SSA are performed on the 121st range cell, and Figure 2b shows the eigenvalue of the self-covariance matrix of the time-delay matrix of this range cell. In the figure, the first two eigenvalues are much larger than the latter. The first two eigenvalues correspond to the rigid part of the distance unit, and the rest correspond to the part of the propeller blade of the aircraft. In Figure 2c, solid blue line is the result of pulse compression of rigid body doppler recovered from the first two characteristic values in Figure 2b, and the red dotted line is the result of pulse compression of the original signal. The principal component corresponding to the two eigenvalues are shown in Figure 2d,e. It can be clearly seen that the influence of the moving parts has been removed, so that the drastic change of the eigenvalue can be used as a grouping criterion.

Eigenvalues are grouped as $\Lambda_{D}=\left\{\lambda_{1},\cdots ,\lambda_{I}\right\}$ and $\Lambda_{m}-D=\left\{\lambda_{I}+1,\cdots ,\lambda_{d}\right\}$, for rigid parts and moving parts. The matrix corresponding to them are

(13) $$\begin{array}{cc} X_{D} & =X_{1}+\cdots +X_{I} \end{array}$$

(14) $$\begin{array}{cc} X_{m-D} & =X_{I+1}+\cdots +X_{L} \end{array}$$

### 4.4. Reconstruction

Suppose $L^{*}=\min\left(L,k\right)$, $K^{*}=\max\left(L,k\right)$, time series corresponding to $X_{D}$ is $S_{D}=\left\{s_{D,0},and\cdots ,s_{D,j},\cdots ,s_{D,M-1}\right\}$, $x_{D,q,p}$ is the element in $X_{D}$. Echo time series of target’s rigid part is

(15) $$S_{D,j}=\left\{\begin{array}{cc} \frac{1}{j+1}\sum_{i=1}^{j+1}x_{D,i,j-i+2}^{*}\; & 0\leq j<L^{*}\\ \frac{1}{L^{*}}\sum_{i=1}^{L^{*}}x_{D,i,j-i+2}^{*}\; & L^{*}-1\leq j<K^{*}\\ \frac{1}{M-j}\sum_{i=j-K^{*}+2}^{M-K^{*}+1}x_{D,i,j-i+2}^{*}\; & K^{*}\leq j<M \end{array}\right.$$

where $x_{D,q,p}^{*}=\left\{\begin{array}{cc} x_{D,q,p} & L<K\\ x_{D,p,q}^{*} & L\geq K \end{array}\right.$. Similarly, m-D information can be extracted from the original data without loss.

In the above process, grouping is the vital step, which determines the integrity of m-D information extraction directly. In ISAIL imaging, the influence of the moving parts on different distance units is different, so the signals can be adaptively grouped by the following process:

Step 1: Normalize the eigenvalue. Let $\delta_{i}$ denote the difference between two adjacent eigenvalues of $\lambda_{i}$ and $\lambda_{i+1}$.

Step 2: Select proper threshold ρ and step size *l*. $\delta_{i}>\rho$ will be seemed as drastic change of the eigenvalue, elements before the *i*th eigenvalue are treated as a group. There are multiple scattered points in the rigid body part of the target, and the scattered points in the same region usually show the same trend, while the scattered points in different regions show different trends, and the corresponding characteristic values will change dramatically. For this reason, step size *l* is set. After finding $\delta_{i}<\rho$, judge the situation of $\delta_{i+1}\;\delta_{i+l}$ backward. When all of them are less than ρ, all the eigenvalues after i+1 are considered as a group, which are all the eigenvalues corresponding to the moving part of the target.

**Figure 3:**
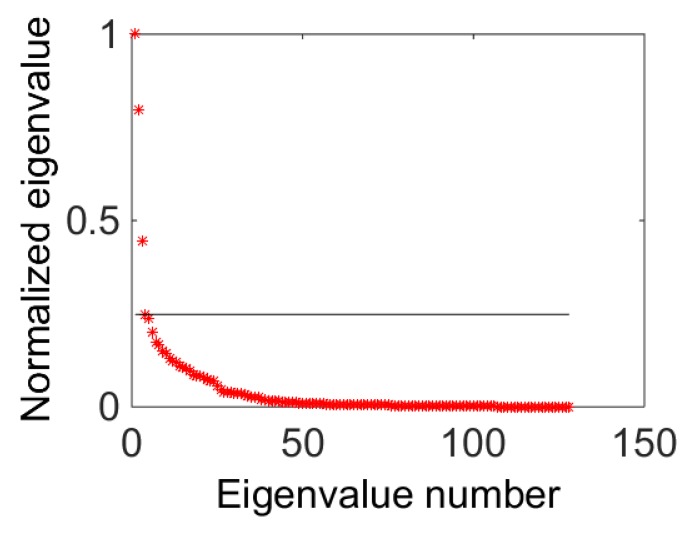
Grouping threshold and step size selection of SSA.

**Figure 4:**
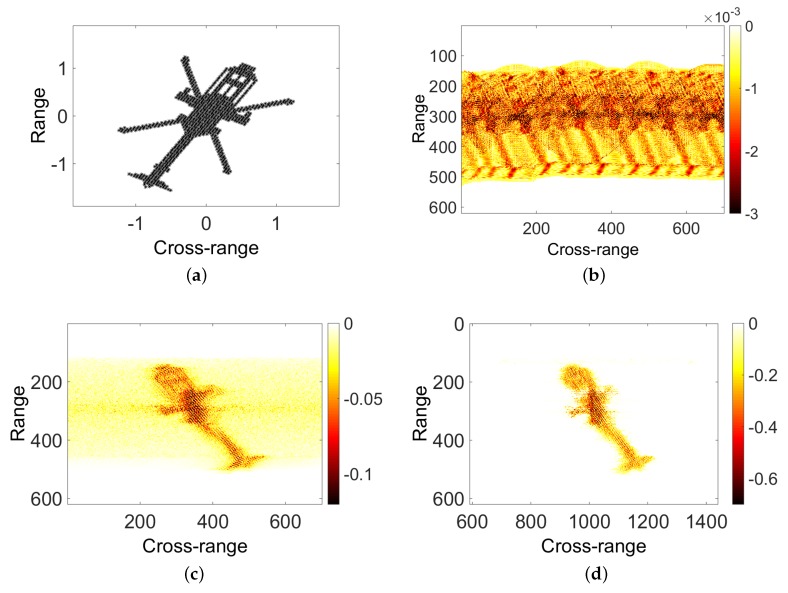

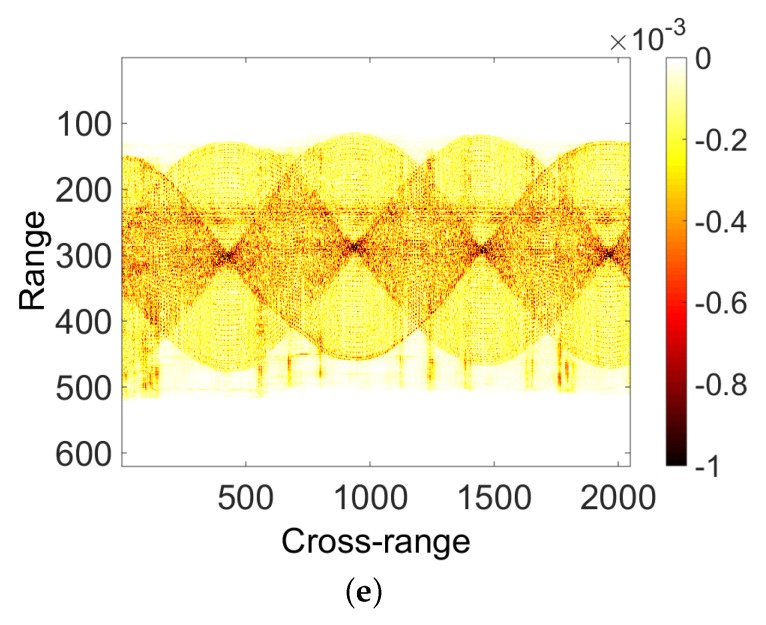
Simulation of helicopter model: (**a**) helicopter model; (**b**) result of distance pulse compression; (**c**) original direct imaging; (**d**) SSA result of helicopter body; and (**e**) SSA result of micro-Doppler signal.

The ISAIL data of An-26 aircraft is illustrated as an example (Figure 3), which is the data analysis result of the 126th range cell. In Figure 4, the straight line is the position of the threshold value ρ; the characteristic value greater than ρ (including ρ value) corresponds to the rigid body part of the aircraft, and the rest correspond to the moving parts such as the aircraft propeller. In this analysis, ρ=0.15,l=5. ISAIL imaging is carried out by separating the rigid body part echo signals in Step 2, and the entropy of the imaging results is calculated.

Step 3: Repeat Steps 1 and 2 for the residual signal of the original signal separated in Step 2, and superimposing the separated echo signal of the rigid body on the previously separated echo signal for ISAIL imaging. Calculate the image entropy value and compare it with the previous one until the value changes in a satisfactory range, stopping the iterative process.

## 5. Simulation

To verify the effectiveness of the proposed SSA based m-D feature extraction algorithm, the proposed algorithm was tested through numerical simulation and real ISAIL m-D data. First, ISAIL data were simulated on the helicopter miniaturization model. The simulation parameters are shown in Table 1.

The imaging results are shown in Figure 4. Figure 4a is the helicopter model used in the simulation. Figure 4b is the result of distance pulse compression. Envelope alignment and phase correction were conducted using methods in the literature [17]. It is obvious that the m-D generated by the main rotor interferes with the helicopter’s main echo signal. Figure 4c shows the result of direct imaging without separation of m-D information, and the helicopter rigid-body imaging result is obviously disturbed. Figure 4d is the result of the imaging of the helicopter body after the separation of the m-D information using the SSA method. To improve the accuracy of the separation of two signal components, the processing of the echo signal is interpolated along the cross-range direction. Figure 4e is the micro-Doppler signal isolated from the original signal by the SSA method.

**Figure 5:**
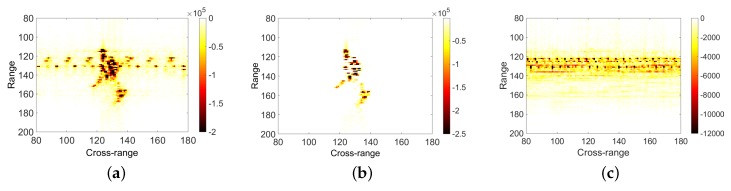
ISAIL result of An-26 by after SSA processing: (**a**) imaging results based on the R-D imaging algorithm; (**b**) SSA result of rigid body part; and (**c**) isolated m-D information.

Second, The SSA-based m-D feature extraction algorithm was validated through the real data of An-26 aircraft. The imaging results are shown in Figure 5. Figure 5a shows the imaging results based on the R-D imaging algorithm, with obvious m-D interference between the range cell 118 and 142. In Figure 5b, the data are processed by SSA algorithm. After separating the echo signal of rigid body part and that of moving part such as propeller, the imaging results of R-D algorithm are used. Figure 5c shows the isolated m-D information.

In the above simulation experiments and the real data test, the SSA-based m-D feature extraction algorithm is effective in the processing of ISAIL target imaging.

## 6. Conclusions

In general, the target of ISAIL imaging is considered to be a rigid body, but some targets such as propeller aircraft have moving parts, and m-D information is added to the echo. On the one hand, this m-D information may be seen as strong horizontal bar interference in ISAIL imaging, which will cause certain difficulties for ISAIL target recognition. On the other hand, if m-D information can be extracted without loss, more target motion information could be obtained.

Aiming at these problems, this paper proposes a method based on SSA theory for non-rigid target micro-Doppler effect analysis. The SSA method decomposes target echo signals; echoes of the rigid parts on target are separated from the m-D information caused by the moving parts. Minimum entropy criterion is combined to obtain a clear ISAIL image. Simulations indicate that the proposed algorithm can extract the rigid parts from the target echo signal, and has good ability anti m-D effect.

## Figures and Tables

**Table 1:** Simulation parameters.

Parameter/Variable	Value	Parameter/Variable	Value
Center wavelength	$10.6\times 10^{-6}$ m	Target size	2.5 m ×2.5 m
Transmitting bandwidth	$20\times 10^{9}$ Hz	Velocity	100 m/s
Pulse repetition frequency	$7\times 10^{3}$ Hz	Main rotor speed	10 r/s
Coherent accumulation time	0.1 s	Route angle relative to radar ray	$30^{\circ }$
Transmitting width	1μs	Imaging distance	10 Km
Range sampling points	620	Cross-range sampling points	700
